# Premature Deaths in Brazil Associated With Long‐Term Exposure to PM_2.5_ From Amazon Fires Between 2016 and 2019

**DOI:** 10.1029/2020GH000268

**Published:** 2020-08-01

**Authors:** M. O. Nawaz, D. K. Henze

**Affiliations:** ^1^ Department of Mechanical Engineering University of Colorado Boulder Boulder CO USA

**Keywords:** adjoint, modeling, PM_2.5_, Amazon, fires, biomass

## Abstract

Amazonian deforestation from slash‐and‐burn practices is a significant contributor to biomass burning within Brazil. Fires emit carbonaceous aerosols that negatively impact human health by increasing fine particulate matter (PM_2.5_) exposure. These negative effects on health compound the already detrimental climatological and ecological impacts. Despite high biomass burning emissions in Brazil and the international attention drawn by the relaxation of Amazon protections in 2019, little is known about the health impacts from PM_2.5_ exposure attributable to these fires. We estimate PM_2.5_‐related premature deaths in Brazil associated with biomass burning, focusing on temporal, interannual, and spatial trends. We find that during the fire season of 2019, 4,966 (2,427, 8,340) premature deaths were attributable to fire emissions making up 10% (5, 17) of all PM_2.5_‐related premature deaths in Brazil. Between the 2019 and 2018 seasons, fire emissions increased by 1.37 Tg (1.00, 2.18) or 115% (60, 201), which was responsible for an increase in health impacts of 2,109 (965, 3,623) premature deaths or 74% (54, 98). Biomass burning emissions throughout Brazil contribute significantly to premature deaths, with the largest burning events occurring in northwestern Brazil. The impact of fires on PM_2.5_‐related premature deaths is highest in heavily populated regions despite their fires being 1 to 2 orders of magnitude smaller than the largest burning events. Results from this study characterize the extent to which elevated PM_2.5_ exposure levels owing to fires affect public health in Brazil and present an additional, public health‐focused, support for increased Amazon protections.

## Key Points

- The severe health effects of biomass burning in Brazil compound the detrimental climatological and ecological impacts
- Brazilian biomass burning emissions and health impacts increased by 115% and 74%, respectively, between 2018 and 2019
- Locations of fires were equally or more impactful to public health in Brazil than emission magnitudes

## 1 Introduction

Biomass burning is a major concern to environmental health and sustainability. In addition to detrimental climatic (Z. Liu et al., 2019; Randerson et al., 2006) and ecological (Valderrama et al., 2018) impacts, burning can release large quantities of pollutants that negatively affect public health (Haikerwal et al., 2015). Emitted species include black (BC) and organic (OC) carbonaceous aerosol (Tang & Arellano, 2017) that are primary components of fine particulate matter (PM_2.5_), a pollutant associated with an increased risk of premature death (Burnett et al., 2014) and ranked as the sixth leading mortality risk factor globally in 2015 (Cohen et al., 2017; Forouzanfar et al., 2016). Particles emitted from less severe fires at low injection heights are transported short distances before deposition occurs; however, more severe fires emit particles that are lofted higher and thus transported farther, affecting populated regions downwind and leading to higher exposure (Koplitz et al., 2016). Over 60% of Brazil's land is classified as either evergreen broadleaf forest or savannah according to MODIS Land Cover Type (Friedl et al., 2010). Evergreen broadleaf forests have among the highest emission factors for OC and BC and highest fuel loadings (Akagi et al., 2011) with savannah emission factors and fuel loadings being lower. Higher fuel loadings and emission factors lead to larger and more severe fires that emit more carbonaceous aerosols.

Health impact assessments of biomass burning emissions have been conducted globally (Johnston et al., 2012; Watts et al., 2019), as well as regionally for specific fires. In Europe (Kollanus et al., 2017), 828–1,342 all‐cause nonaccidental premature deaths occurred in 2008 directly attributable to vegetation fire‐oriented PM_2.5_. Mortality increased up to 92% in the United States following large wildfire exposure events (Liu et al., 2015) with an estimated 57 million people affected by smoke waves, defined to be two or more consecutive days with high wildfire‐specific PM_2.5_, between 2004 and 2009 in the Western United States (Liu et al., 2016). In the United States, fire‐related PM_2.5_ was responsible for 17,000 premature deaths per year in the early 21st century (Ford et al., 2018). During the Indonesian peat burning season, burning practices lead to 36,000 premature deaths per year and the extreme 2015 burning season led to exposures causing 100,300 premature deaths (Kim et al., 2015; Koplitz et al., 2016; Marlier, DeFries, Kim, Gaveau, et al., 2015; Marlier, DeFries, Kim, Koplitz, et al., 2015).

Specific to South America, between 1997 and 2006 landscape fire smoke contributed 10,000 premature deaths on average per year with smoke during an El Niño year (September 1997 to August 1998) contributing 19,000 premature deaths and smoke during a La Niña (September 1999 to August 2000) contributing 11,000 premature deaths (Johnston et al., 2012). Across South America, between 2002 and 2011, deforestation fires were responsible for, on average, 2,906 premature deaths from cardiopulmonary disease and lung cancer (Reddington et al., 2015). Pregnant women exposed to elevated PM_2.5_ from agricultural burning had higher rates of low birth weight babies in one Brazilian state (Cândido da Silva et al., 2014; Reid et al., 2016).

In 2019 the Instituto Nacional de Persquisas Espaciais (INPE) of Brazil identified 118,563 individual fires between July and September (INPE, 2019)—one of the higher fire counts observed in the last decade across this 3 month period. This abundance of fires, in addition to the relaxation of certain protections of the Amazon by the Brazilian government (de Area Leão Pereira et al., 2019), led to the 2019 season garnering international attention (Andreoni & Londoño, 2019). Much of this attention has focused on the climatic and ecological impacts of the fires while the pollution and health effects have remained relatively unstudied. Additionally, although the spatial distribution of fires themselves are relatively well known given satellite‐based detection systems (INPE, 2019), it is less clear how the spatial distribution and magnitude of fires relate to their public health impacts, and the relative significance of fire count and intensity in the context of interannual variability of burning events. Past environmental policy interventions have considered the cobenefits of improved air quality and better public health when targeting other climate or pollution issues (Scovronick et al., 2019). Similarly, the potential cobenefits of reduced fire emissions for health could be considered in addition to the climatic and ecological benefits of reducing slash‐and‐burn farming when making related policy decisions.

In this study we estimate PM_2.5_‐related premature death in Brazil owing to Brazilian biomass burning during the fire seasons of the last 4 years. This is done by combining emission estimates with integrated exposure response (IER) relationships from the 2016 Global Burden of Disease (GBD) study (Vos et al., 2017) and source‐receptor relationships from a chemical transport model (GEOS‐Chem adjoint (Henze et al., 2007). We use biomass burning emission estimates from two sources: the Quick Fire Emissions Dataset (QFED) (Darmenov & da Silva, 2015) and the Fire Inventory from NCAR (FINN) (Wiedinmyer et al., 2011) to account for uncertainties in emission inventories. Previous comparisons (Pan et al., 2019) of OC emissions in 2008 in a region similar to our study domain found that estimates ranged from 3.3 to 6.0 Tg yr^−1^, for FINN and QFED, respectively. One comparison of five emission inventories for all of South America found that QFED had the highest OC and BC emissions with FINN having the third highest for both species over 2003–2016 (T. Liu et al., 2020). Another study compared aircraft measurements and in situ monitors to simulated concentrations of BC and OC using FINN emissions in the Amazon. (Reddington et al., 2019). Aircraft observation normalized mean biases ranged from −0.13 to 0.56 for BC in the western Amazon across two campaigns and −0.25 to 1.21 for OC. In the eastern Amazon BC and OC had larger negative biases of −6.11 and −3.14, respectively. Between 2002 and 2012, in the Amazon, 1.24 Tg of OC and 0.14 Tg of BC were emitted as estimated by FINN.

We employ adjoint modeling because determining the air quality and health impacts of fire emissions across different days and locations would be computationally intensive without its use; instead of performing thousands of model simulations with emissions perturbations for every fire and every day, we can run an adjoint model once to obtain the sensitivity of PM_2.5_ exposure in Brazil to all individual fires simultaneously. This approach includes the role of atmospheric transport and deposition in the assessment of the contributions of these emissions to exposure rather than an analysis of only the emission magnitude or frequency such as considered in the 2019 Lancet Countdown (Watts et al., 2019). This modeling framework has previously been used to rapidly assess the air quality and health impacts of peat burning (Kim et al., 2015; Koplitz et al., 2016; Marlier, DeFries, Kim, Gaveau, et al., 2015; Marlier, DeFries, Kim, Koplitz, et al., 2015). We quantify the deaths prematurely caused by fires and focus on the spatial and interannual variability of the health impacts from different burning locations (Nawaz & Henze, 2020).

## 2 Methodology

### 2.1 Fire Emissions From QFED and FINN

In this analysis, we use two distinct fire emission data sets: FINNv1.5 (Wiedinmyer et al., 2011) and QFEDv2.4 (Darmenov and da Silva, 2015). These fire emission inventories represent two distinct methodological approaches for estimating biomass burning and are used to bound emission‐related uncertainty in our analysis. For FINN, satellite observations of land cover and active fires are combined with bottom‐up biomass characterizations as nonuniform points down to 1 km^2^ spatial resolution representing emissions from individual or clustered fires at a daily temporal resolution. Through satellite observations, fires are identified and the areas burned are estimated at a given time and location. Then these areas are combined with biomass loading, fraction of biomass burned and speciated emission factors from that same location to estimate emissions. In this study, we sum all of the individual emission point sources within each grid cell and divide by the area of that grid cell to create emission fluxes at the 2° × 2.5° resolution of our modeled sensitivities.

QFED biomass burning emissions are estimated using the fire radiative power (FRP) approach which combines the MODIS FRP product, the instantaneous rate of fire radiative energy, emission factors, and area density to provide high‐resolution globally estimated emissions. These emissions are provided as fluxes at 0.1° × 0.1° resolution; we regrid finely resolved QFED emissions to the coarser resolution of our modeled sensitivities by averaging the fluxes of all fine‐resolution grid cells contained within a coarse cell.

### 2.2 Adjoint Sensitivity Analysis

**Figure 1:**
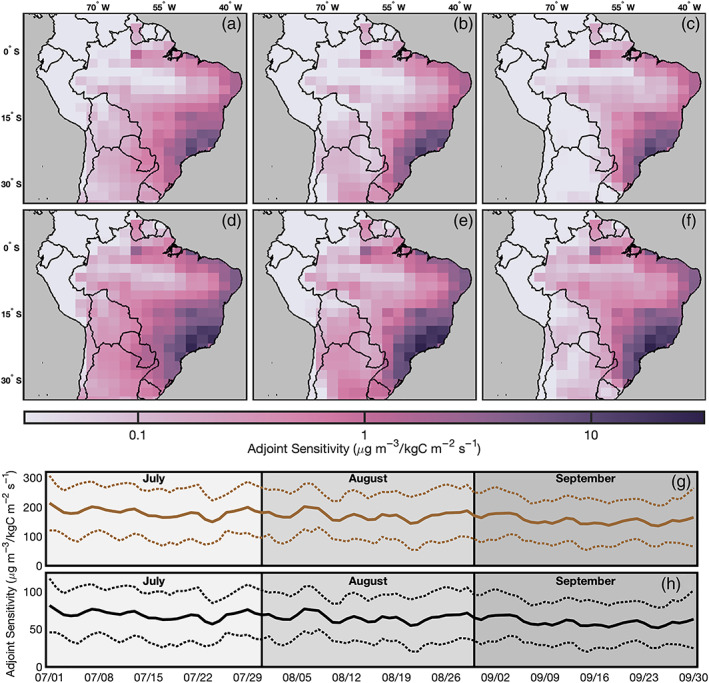
Daily adjoint sensitivities for the first day of each month for BC in July (a), August (b), and September (c) and for OC in July (d), August (e), and September (f). Spatial mean adjoint sensitivity across Brazil for OC (g) and BC (h) with 95% confidence intervals from uncertainty in averaging sensitivities between 2009 and 2011.

Here we use the GEOS‐Chem adjoint model (Henze et al., 2007) v35 for source apportionment at the 2° × 2.5° horizontal resolution. The adjoint code originally corresponded to v8‐02‐01 of the GEOS‐Chem forward model and has been updated with forward model updates and fixes up to version 10. We refer to the standard GEOS‐Chem model as the forward run and the gradient calculation as the adjoint run. We choose to employ adjoint modeling, as opposed to finite difference forward modeling, since we are considering the impact of ~821,376 unique combinations of emissions, which would be prohibitively expensive to explore via direct zero‐out simulations. The current version of the GEOS‐Chem adjoint model is limited in its simulation of PM_2.5_ because it does not include secondary organic aerosol formation or fugitive dust. Using the HTAPv2 (Janssens‐Maenhout et al., 2015) inventory for anthropogenic emissions of OC and BC, we conduct 12 adjoint runs across 2009, 2010, and 2011 simulating the tropospheric transport and development of these species using GEOS‐5 meteorological fields from the Goddard Earth Observation System (GEOS) DAS of the NASA Global Modeling and Assimilation Office (GMAO). For each year, we conduct runs for July, August, and September including a 1 month spin‐up period before performing each individual 1 month run and outputting daily sensitivities. We perform three additional runs for October to account for sensitivities to emissions at the end of September. We take the average sensitivity across these three years for each day to characterize transport behaviors as opposed to specific meteorologies for application to future years. We present these averaged adjoint sensitivities for BC and OC on the first day of each month in Figure 1. Additionally we present a time series of daily sensitivities in Brazil along with the estimated uncertainty introduced by the averaging of sensitivities across three years.

In sensitivity analyses, adjoint modeling allows for the efficient calculation of the gradient *λ*_*E*_ of a cost function *J* with respect to multiple emission parameters *E*. Generally, the cost function represents some quantity of interest computed from the forward model output. In this study our cost function is annual average population‐weighted downscaled PM_2.5_ in Brazil defined as:

(1) $$J=\frac{\sum_{i\in B}\left(P_{i}\times {\bar{X}}_{I}\frac{\mathrm{sa}t_{i}}{\mathrm{SA}T_{I}}\frac{\mathrm{SA}T_{I}}{{\bar{X}}_{I}}\right)}{\sum_{i\in B}P_{i}}\;$$

Here, *i* refers to spatial indexing at 0.1° × 0.1° resolution, and *I* refers to spatial indexing at the 2° × 2.5° resolution, ${\bar{X}}_{I}$ represents annual averaged PM_2.5_ output from the forward model, *sat*_*i*_ is a satellite‐derived product (Shaddick et al., 2018), *SAT*_*I*_ is this same product averaged at the coarse model resolution, *P*_*i*_ is a fine‐resolution population estimate (CIESIN, 2016) and *B* is the set of all grid cells within Brazil. This cost function is the specific metric suitable for analysis of chronic health impacts of PM_2.5_ exposure.

During the forward run the cost function is calculated using Equation 1. Following this, the adjoint run is conducted returning gradients of the cost function to emissions of black and organic carbonaceous aerosol:

(2) $$\lambda_{I,s}=\nabla_{E_{I,s}}\;J=\frac{\partial J}{\partial E_{I,s}}\;$$

where *λ*_*I*,*s*_ is the sensitivity of the cost function to emissions from species *s* at location *I* and *E*_*I*,*s*_ refers to the emissions of species *s* at location *I*. From our adjoint runs we obtain the sensitivities of our cost function to emissions of OC and BC and then take the average across all three years for each month. We do this to avoid extreme meteorology from any individual year since we are applying these sensitivities to estimate biomass burning contributions in an entirely different time period.

We regrid the fine resolution fire emissions from QFED and FINN to the resolution of the GEOS‐Chem sensitivities and then combine them with our monthly sensitivities to estimate the contribution of the fire inventory biomass burning emissions to population‐weighted PM_2.5_. From this calculation we determine the amount that daily emissions from each individual grid cell contribute to downscaled population‐weighted PM_2.5_ in Brazil:

(3) $$dJ_{I,d}=\sum_{s=\mathrm{OC},\mathrm{BC}}\left(\lambda_{I,s,d}\times E_{I,s,d}\right)\;$$

Here we refer to *dJ*_*I*,*d*_ as the contribution of emissions to annual average population‐weighted downscaled PM_2.5_ at location *I* on day *d*. *E*_*I*,*s*,*d*_ represents the regridded FINN or QFED emissions which have daily *d* temporal resolution and are separated by species *s*.

### 2.3 Health Impact Analysis

We calculate the PM_2.5_‐related premature death following the framework used in the 2016 GBD study. Population data from version four of the Gridded Population of the World (GPWv4) for 2010 (CIESIN, 2016) and age‐stratified GBD mortality rates for the health outcomes of ischemic heart disease (IHD), stroke, chronic obstructive pulmonary disease (COPD), lung cancer (LC), and acute lower respiratory illness (ALRI) are combined with an attributable fraction (AF) defined as:

(4) $$\mathrm{AF}\left(z\right)=\frac{\mathrm{RR}\left(z\right)-1}{\mathrm{RR}\left(z\right)}\;$$

Here, *z* is the population‐weighted PM_2.5_ concentration which can represent either the total population‐weighted PM_2.5_ or this same total with the fire contribution, *dJ*_*I*,*d*_, removed as discussed at the end of this section. We denote *RR* as the relative risk which is the ratio of the risk of a health outcome occurring in an exposed population versus an unexposed population. In this case the exposure is to PM_2.5_. We estimate the relative risk using integrated‐exposure response functions from the GBD 2016 study:

(5) $$\mathrm{RR}\left(z\right)=\mathrm{IER}\left(z\right)=1+\alpha \times \left(1-e^{-\beta {\left(z-z_{\mathrm{cf}}\right)}^{\gamma }}\right)$$

where *α*, *β*, and *γ* are parameters dependent on the health outcome and age, *z* is the PM_2.5_ exposure, and *z*_*cf*_ is the counterfactual concentration. Any concentration below *z*_*cf*_ is given a relative risk of one indicating that these concentrations have no increased risk for a health outcome. With the relative risks calculated, we can estimate the associated premature deaths as:

(6) $${\left(\text{Premature Mortality}\right)}_{O}=P\times \mathrm{BM}R_{O}\times \mathrm{AF}\left(z\right)$$

The premature death of health outcome *O* is equivalent to the population of Brazil *P* combined with the national Brazilian baseline mortality rate of the same health outcome *BMR*_*O*_ and the attributable fraction of PM_2.5_ exposure *z*. We use age‐specific mortality rates, population and IER parameters and sum over all age groups above 25 in the 5‐year brackets used in GBD 2016. We calculate this for all five aforementioned health outcomes and add these to approximate the premature death associated with the PM_2.5_ exposure.

To calculate the premature deaths specific to biomass burning emissions we must consider both the total annual PM_2.5_ exposure and the contribution of biomass burning emissions to the PM_2.5_ exposure. We estimate the total PM_2.5_ exposure using satellite derived estimate (Shaddick et al., 2018) for 2010 and then remove the biomass burning emission contributions *dJ*_*I*,*d*_ which refer to the amount of PM_2.5_ exposure attributable to emissions at location *I* on day *d*. By removing these contributions we approximate the PM_2.5_ exposure that would exist if no fires occurred at location *I* on day *d*. We calculate the total premature deaths for both the base PM_2.5_ exposure and the fireless PM_2.5_ exposure for every grid cell and day and take the difference to determine premature deaths contributed by biomass burning emissions from each grid cell and day.

When compared to a previous global burden of disease study (Cohen et al., 2017), the population weighted PM_2.5_ in Brazil estimated in this study (11.2 ug/m3) was comparable (11.4 ug/m^3^). When removing fire‐related PM_2.5_ exposure from total PM_2.5_ exposure we are ignoring the differing impact of composition on health which introduces uncertainty into the assessment. Additionally, increased population in Brazil since 2010 would lead to increases in the exposed population; this would indicate an underestimation of premature deaths in our analysis.

### 2.4 Uncertainty Analysis

Uncertainty in our final premature death estimates are introduced primarily from three sources: GEOS‐Chem modeling, Amazon biomass burning emission inventories, and integrated exposure response functions.

Aerosol modeling in GEOS‐Chem is relatively robust; however, simulated aerosols do not match observed results. In order to account for this, we utilize the downscaling method previously described to incorporate PM_2.5_ estimates from satellite aerosol optical depth observations. The satellite‐derived data set is statistically strong when compared to in situ observations (Shaddick et al., 2018) (*R* ^2^ = 0.90). We use the satellite‐derived product for 2010; however, using a more recent year, 2015, we find premature death increases on average by 14% in a given month. Since not all years of study have a satellite‐derived product, we opted to use a year consistent with the meteorology; however, this likely results in an underestimate of premature death. Additionally, by using 3 year averaged adjoint sensitivities, we introduce error by not using the study years meteorology. To account for the uncertainty in the sensitivities, we calculate the standard deviation of our sensitivities for each day. We then propagate this standard deviation into our perturbation concentrations (all of the steps involved in this, outlined prior, are linear) and then into our relative risk equation as:

(7) $$\sigma_{\mathrm{RR}}^{2}={\left(\frac{\mathrm{dRR}}{\mathrm{dz}}\right)}^{2}\times \sigma_{z}^{2}$$

From this standard deviation we calculate the 95% confidence interval attributable to the adjoint sensitivity averaging uncertainty.

Uncertainty inherent to the biomass burning emission inventories are discussed in their associated papers (Darmenov and da Silva 2015; Wiedinmyer et al., 2011) as well as other works (T. Liu et al., 2020; Pan et al., 2019). We use two separate inventories in order to better account for this uncertainty. When presenting emission results and using emissions for health impact calculations we take the average for each year and use the actual emissions estimates as bounds for the emission uncertainty.

There are large uncertainties in the integrated exposure response functions used to estimate the risk from exposure to PM_2.5_. Here we use 1,000 parameter draws from the GBD 2016 IER curves and calculate the risk and subsequent premature deaths for each of the 1,000 distinct sets. We take the mean of the difference ( ${\bar{M}}_{d}$) between total premature death and the “fireless” total premature deaths for all 1,000 unique IER parameter sets and calculate the 95% confidence interval of the difference of the means treating them as matched samples:

(8) $${\mathrm{CI}}_{0.95}=\left({\bar{M}}_{d}\right)\pm 1.96\times \frac{s_{d}}{\sqrt{n}}\;$$

where *s*_*d*_ is the standard deviation of the difference and *n* is the number of data points. By treating the values as matched samples, as opposed to independent samples, we assume that the parameters of the IER are unaffected by the contribution of biomass burning emissions. Though we consider all 1,000 unique parameter combinations, we assume that the risk from total PM_2.5_ exposure and the risk from the PM_2.5_ exposure with biomass burning removed would be calculated using the same set of parameters in each of the 1,000 cases. This is consistent with the GBD model framework since relative risks are fit to the IER parameters using a Bayesian framework, where in each case the IER function outputs estimates of relative risks for inputs of all values of PM_2.5_ exposure (Cohen et al., 2017).

We calculate the confidence intervals for both emissions data sets and every year of the study. When presenting the error bounds of premature death estimates we combine the bounds of the emission data sets along with the uncertainty in the IER functions and adjoint sensitivities. To consider emission, IER, and adjoint uncertainty in the uncertainty range, we first determine the emission data sets that have the lower and higher estimate for that year and then take the lower and upper bound of their respective confidence intervals to determine the final bounds.

## 3 Results

### 3.1 Biomass Burning Emission Trends

**Figure 2:**
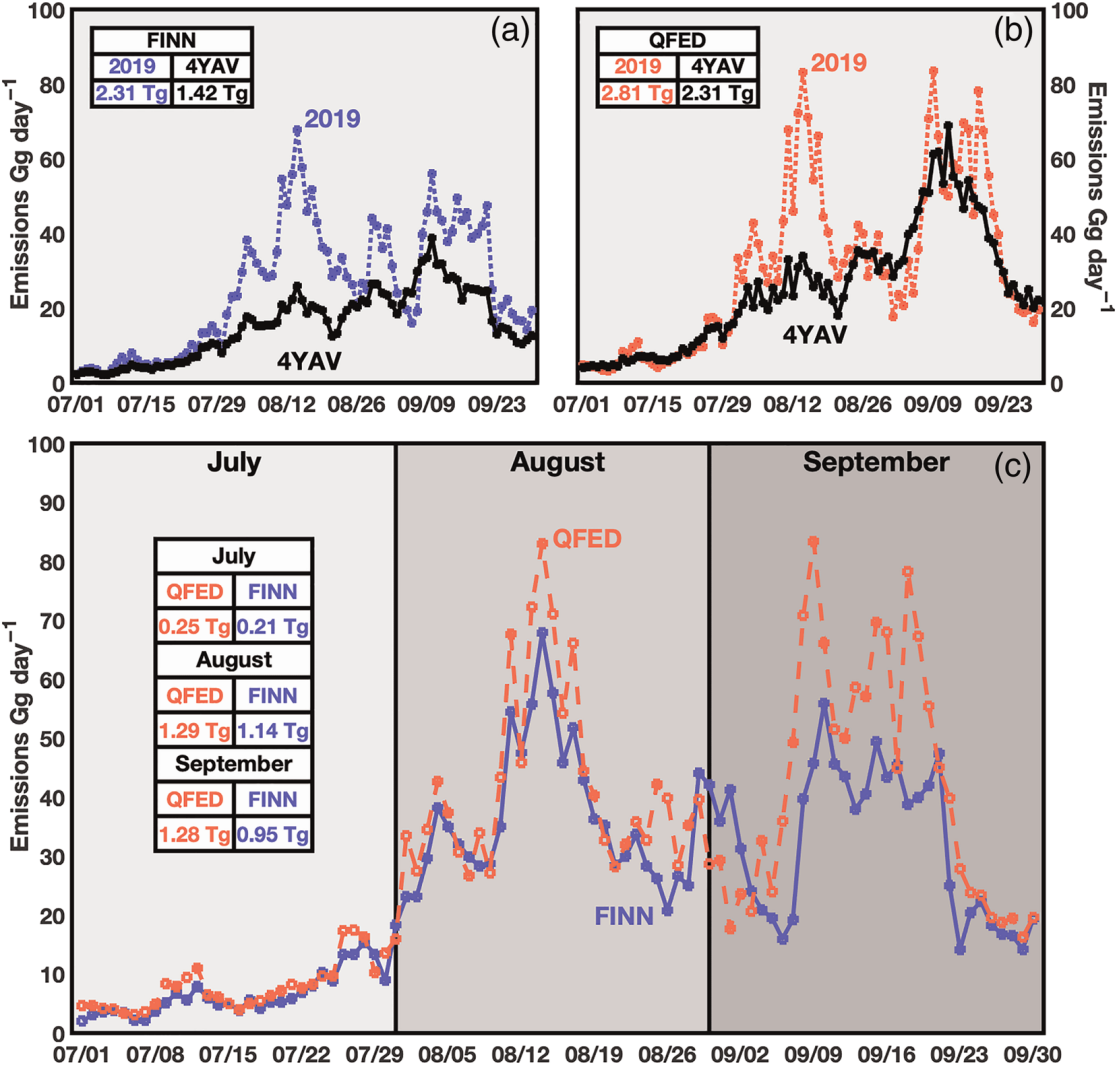
Comparison between 2019 and 4 year average (4YAV) of 2016–2019 daily carbonaceous aerosol emissions from (a) FINN and (b) QFED. Seasonal total emissions are listed in each respective color. (c) Comparison between FINN and QFED in 2019. Monthly emission totals are reported in the legend.

We first evaluate the magnitude of biomass burning emissions from 2016–2019 using two different inventories. By comparing 2019 emissions with the 4 year average (4YAV), we find that both FINN (Figure 2a) and QFED (Figure 2b) show elevated seasonal emissions of 163% and 122%, respectively, compared to previous years. The most noticeable differences occur in August and September when 2019 values are up to 262% higher than the 4 year average values (FINN, 11 August). We compare the two data sets directly in 2019 (Figure 2c) and find that QFED emissions were higher than FINN in 2019 by 25% on average. This is the smallest percent difference of all four years; QFED emissions were 129%, 95%, and 72% higher than FINN in 2016, 2017, and 2018, respectively. In all four years, July had significantly lower emissions than August and September. The total national 4 year average carbonaceous aerosol emissions across FINN and QFED and all fire types between July and September is 1.86 Tg yr^−1^ (1.42, 2.31).

### 3.2 Temporal Trends in Biomass Burning Attributable Premature Mortality

**Figure 3:**
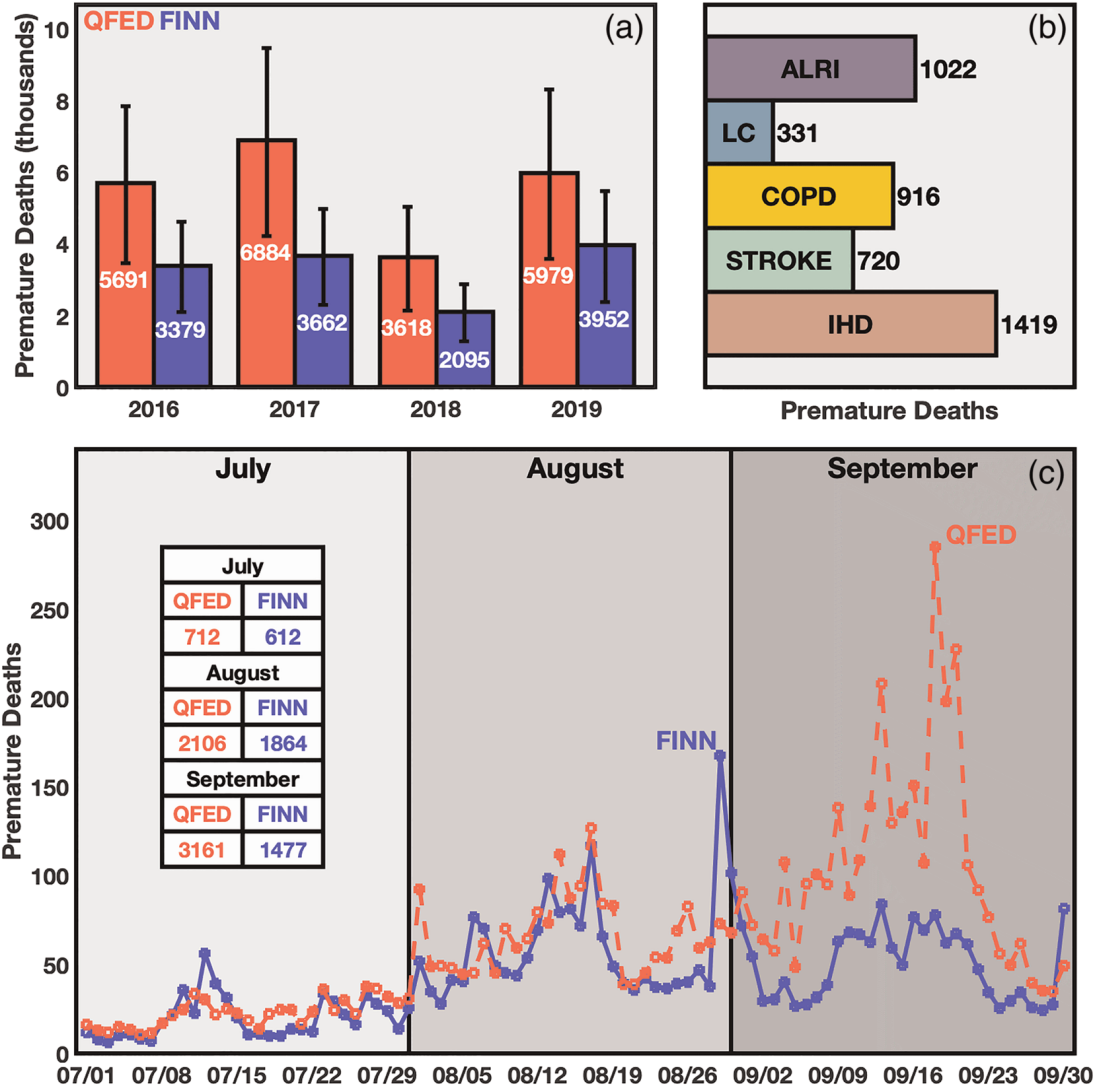
Contributions to annual average PM_2.5_‐related premature deaths by year and emissions data set (a). Contributions of each health outcome to total estimated premature deaths for all years and data sets averaged (b). Time series of 2019 contributions to annual average PM_2.5_‐related premature deaths (c).

During the fire seasons, biomass burning emissions led to elevated concentrations of PM_2.5_ and caused severe health impacts. Quantified health impacts vary by emission inventory estimates and year (Figure 3a), with fire emissions causing 4,407 (1,302, 9,550) premature deaths per year on average. Uncertainty ranges presented here include adjoint, emission and IER uncertainties and are discussed in detail in the methodology section. Between 2016 and 2019 the QFED‐derived premature death estimates were usually greater than the FINN‐derived estimates; however, both had similar day‐to‐day variability. The 2019 season had the second highest number of biomass burning attributable premature deaths when averaged across both emission data sets, with 4,966 (2,426, 8,380) premature deaths occurring in 2019 and 5,273 (2,344, 9,550) occurring in 2017. Compared to 2018, premature deaths attributable to biomass burning in 2019 were 74% (54, 98) greater.

In both the temporal and interannual analysis discussed here we find a common result: There is a clear difference between the magnitude of emissions and the magnitude of health impacts. Days with high emissions can contribute relatively little to premature deaths from PM_2.5_ exposure. More specifically, in September the difference between QFED and FINN in magnitudes of emissions (Figure 2c) is significantly lower than the difference in health impacts (Figure 3c). This result can be attributed to QFED emissions generally being greater than FINN emissions in locations that were more sensitive to PM_2.5_ exposure, as further discussed in section 3.4.

The health outcome most responsible for biomass burning attributable premature deaths was IHD (Figure 3b), accounting for 32% of all premature deaths averaged across our 4 year study period and both QFED and FINN. However, ALRI contributed significantly to premature deaths as well accounting for 23% of all premature deaths. Generally, when examining total PM_2.5_‐related premature deaths IHD is found to be the largest contributor (Burnett et al., 2014), which we also find when calculating total premature deaths here. When calculating just the fire emission contribution, we remove a portion of the PM_2.5_ exposure associated with fire emissions. This removal of PM_2.5_ affects health outcomes differently depending on the relationship between increased risk of premature death and PM_2.5_ exposure. IHD and ALRI are all highly sensitive to changes in PM_2.5_ exposure at the annual PM_2.5_ exposure level for Brazil explaining their similar overall contributions to premature death.

Monthly patterns in biomass burning attributable premature deaths (Figure 3c) match with emissions (Figure 2c). July emissions contribute the least to premature deaths while premature death contributions peak in August from FINN estimates and in September from QFED.

### 3.3 Spatial Trends in Biomass Burning Attributable Premature Mortality

Though fires appear throughout much of Brazil they are not equally impactful; biomass burning emissions more directly upwind of densely populated regions result in greater PM_2.5_ exposure and a larger mortality burden than those in more remote regions. Here we use mortality burden to refer to “the premature deaths attributable directly to emissions from biomass burning averaged across both emission data sets.”

**Figure 4:**
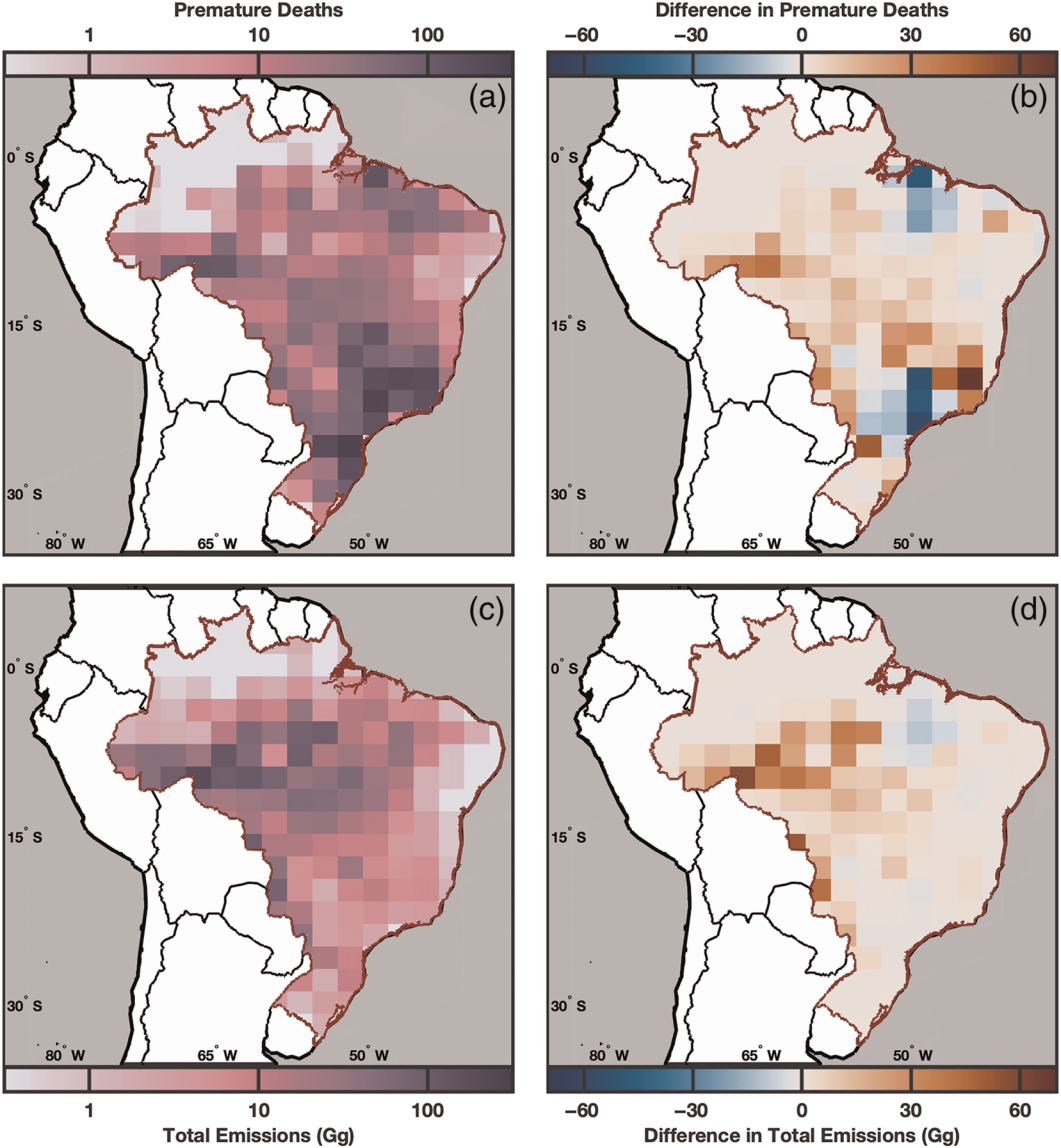
Spatial distribution of raw contributions to annual average PM_2.5_‐related premature death in 2019 (a)**.** Difference between 2019 mortalities and 4 year average (b). Emissions averaged between two data sets for 2019 (c). Difference in emissions between 2019 and 4 year average (d).

There are large spatial variations in the mortality burden (Figure 4a) throughout our study. The region that has the most impactful emissions on the mortality burden is the heavily populated southeastern region of Brazil which includes the cities of Sao Paulo, Rio de Janeiro, and Curitiba. Other regions with emissions that contribute significantly to the mortality burden include western Brazil by the border of Bolivia and northern Brazil near the Amazon River estuary. Emissions from Central Brazil contribute very little to the mortality burden.

We compare the mortality burden spatial distribution from 2019 to a 4 year averaged distribution (Figure 4b) as an absolute difference. Emissions during the 2019 fire season had above average contributions to the mortality burden in multiple regions including much of western Brazil, directly north of Rio De Janeiro and the densely populated region near Brazil's borders with Paraguay and Argentina. Below average mortality burden contributions occurred in the area surrounding Sao Paulo and throughout much of northern Brazil. Results presented here combine the two emission data sets though there are minor differences in the spatial distributions of FINN and QFED‐derived results discussed in the next subsection.

In both the temporal and interannual results we find that the magnitudes of premature deaths are not solely dependent on the magnitude of emissions; this pattern is also observed in the spatial distribution. When comparing emissions (Figure 4c) to the mortality burden contributions (Figure 4a) in 2019 it is clear that the areas with the largest mortality burden contributions are not necessarily the areas of highest emissions. Regions with larger fires, denoted as areas that emit over 50 Gg of carbonaceous aerosol per season, only contributed 679 premature deaths or 14% of the mortality burden. Similarly, regions with high premature death contributions, denoted as areas that contribute over 50 premature deaths per season, which when combined contributed 3,142 premature deaths, only emitted 728.6 Gg or 28% of the total carbonaceous aerosol emissions.

The spatial distribution of 2019 emissions differed from the 4 year average emissions (Figure 4d) with increases occurring in western Brazil, an area of mostly evergreen broadleaf forests, and decreases occurring in northern Brazil, an area of mostly savannas. When emission differences are compared to changes in the mortality burden distribution (Figure 4b) we find that some of the largest changes in premature death occur in areas that have low (<5 Gg) changes in emissions. These results emphasize the importance of considering the transport of smoke from fires to exposure in populated areas as distinct from the magnitude of the fire emissions alone. A small fire upwind of a population dense region can increase PM_2.5_ exposure more than a much larger fire in a more remote region. Modeled transport years should thus be representative of transport behaviors in our study period. We compare wind vector fields between modeled years and emission years in the supporting information and find consistent transport behaviors in high contributing regions.

From our health impact analysis we calculate a total annual PM_2.5_‐related mortality contribution in Brazil of 47,908 deaths in 2010, which is consistent with GBD results (Cohen et al., 2017). We then compare this total annual mortality to fire emission contributions across all years and emission data sets in order to estimate the proportion of Brazilian PM_2.5_‐related premature deaths attributable to aerosols originating from fire emissions. We find that 10% (5, 17) of all annual PM_2.5_‐related premature deaths in Brazil are attributable to fire emissions emitted between July and September. These percentages are only calculated with contributions occurring during our 3 month period of study; in reality fire emission contributions are likely larger.

### 3.4 Differences in Premature Death Contributions Between Emission Data Sets

**Figure 5:**
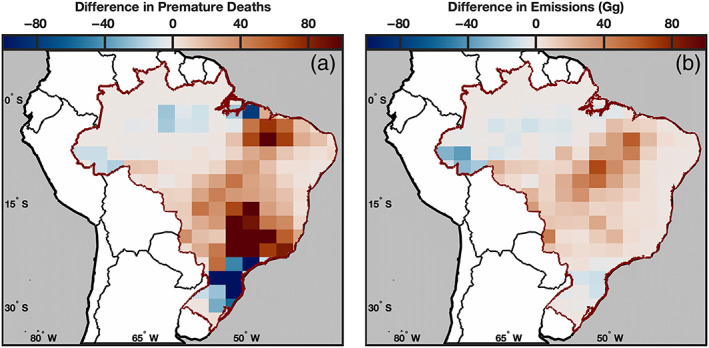
Difference between QFED and FINN in premature deaths (a) and emissions (b) per year over the 4 year average between 2016 and 2019.

Across the entire 4 year average we find that QFED has higher emissions in the more populated central, southeast, and northeast regions of Brazil (Figure 5b). In these regions, QFED emissions are on average 11.3 Gg (0.0, 54.1) larger than FINN in the same area. In contrast, FINN emissions are higher in the more remote northwestern area as well as the densely populated south of Brazil. In these regions, emissions are on average 12.1 Gg (0.0, 65.3) larger than QFED. Emissions closer to populated regions contribute more to PM_2.5_ exposure and subsequently health impacts (Figure 5a). In areas where QFED emissions were larger than FINN they contributed on average 42 (0, 483) more premature deaths, whereas in regions where FINN emissions were larger, they contributed only 30 (0, 444) more premature deaths on average. Despite similar total emissions between the two inventories in 2019, QFED emissions were higher in regions which contributed more to PM_2.5_ exposure and its associated health impacts.

## 4 Discussion

This study quantifies the number of premature deaths due to total biomass burning emissions, as distinct from all PM_2.5_ exposure, in Brazil, for the first time and provides points of comparison for future studies. We find that in 2019 the 4,966 premature deaths contributed by biomass burning emissions between July and September made up 10% of total annual PM_2.5_‐related premature deaths, demonstrating the significance of fire emissions to public health. When compared to Reddington et al. (2015), we estimate greater premature deaths in Brazil in our study period (2016–2019) than were observed from deforestation fires throughout all of South America (2001–2012). The 2019 fire season had increases in both emissions and premature deaths when compared to 2018.

Starting with an approach developed by Koplitz et al. (2016), we introduce a daily climatological (as opposed to annual) emissions‐exposure relationship and combine this with specific fire emission estimates, mortality rates, and population data to rapidly quantify PM_2.5_‐related deaths attributable to biomass burning emissions. This approach characterizes general transport behaviors rather than meteorology from a single year, which may be more broadly applicable for policy regarding future years whose precise meteorology is not yet known.

There are a number of uncertainties and limitations that should be recognized when considering these results. We likely underestimate total biomass burning health impacts since we only consider fires occurring between July and September, only consider health impacts in Brazil, and only consider primary carbonaceous aerosol. These results demonstrate that pollution from biomass burning emissions has a considerable impact on Brazil's air quality. To improve national air quality, both biomass burning and anthropogenic emission levels should be considered. Policies that would result in the increased number or severity of burnings would be detrimental to national health. Protecting the Amazon from future development, specifically near population‐dense regions, could be an effective approach for reducing the number of premature deaths associated with biomass burning emissions.

We conduct three adjoint modeling runs with meteorology from three years, 2009, 2010, and 2011 and average their sensitivities, however, using the meteorological data for the specific years of our study period would be more accurate. Additionally, we use satellite‐derived total PM_2.5_ concentrations for the year 2010. We find that using the 2015 satellite‐derived product increases premature death estimates by 14% on average across the three months and two species. Additionally, our model is relatively coarse at 2° × 2.5° horizontal resolution, so smaller variations in sensitivities are not resolved in our analysis. This resolution error combines highly sensitive cities of small areas with larger less‐sensitive surrounding regions which could lead to an underestimation of PM_2.5_‐related premature death. A finer‐resolution model would be preferable but does not currently exist for the South American domain in the GEOS‐Chem adjoint. Lastly, we only perform the health impact assessment for the population older than 25; this excludes any premature deaths that occur in the rest of the population. If we consider all of these sources of uncertainty, in addition to IER, GEOS‐Chem, and emission uncertainties, it is likely that the results presented here are underestimations of PM_2.5_‐related premature deaths in Brazil.

As fires and their emissions remain a persistent problem in Brazil, a quantification of the health impacts associated with biomass burning provides an additional perspective when considering Amazon protection policy. Compounding the detrimental ecological and climatological effects of fires in Brazil with thousands of premature deaths each year further supports the need for informed, rapid, and effective conservation measures.

## Conflict of Interest

The authors declare no conflicts of interest relevant to this study.

## Supporting information

Supporting Information S1

## Data Availability

All new data generated in this project are stored in the CU Scholar data repository which adheres to the common Enabling FAIR data project (Nawaz & Henze, 2020). Newly generated data include the following: regridded biomass burning emissions between 2016 and 2019, model sensitivities from the GEOS‐Chem adjoint and health impacts.
