# Supplementary material for: Premature Deaths in Brazil Associated With Long‐Term Exposure to PM2.5 From Amazon Fires Between 2016 and 2019
Source: Geohealth. 2020 Aug 1;4(8):e2020GH000268. doi: 10.1029/2020GH000268 (PMC7442537; doi:10.1029/2020GH000268)
Supplement: Supplementary file 1 — Supporting Information S1 [file GH2-4-e2020GH000268-s001.docx]

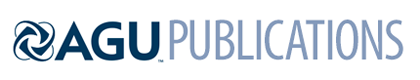


*GeoHealth*

Supporting Information for

**Premature deaths in Brazil associated with long-term exposure to PM_2.5_ from Amazon fires between 2016-2019**

M. O. Nawaz^1^ and D. K. Henze^1^

^1^Department of Mechanical Engineering, University of Colorado Boulder, Boulder, CO, 80301, USA

**Contents of this file**

Figures S1 to S3

Tables S1 to S2

**Introduction**

In this supporting information, two tables and three figures are included. The figures are of monthly wind flow fields across the modeling domain for all emission and modeling years. The tables indicate the percentage of biomass burning emissions occurring during our study period and the emissions and premature deaths contributed by each land type.


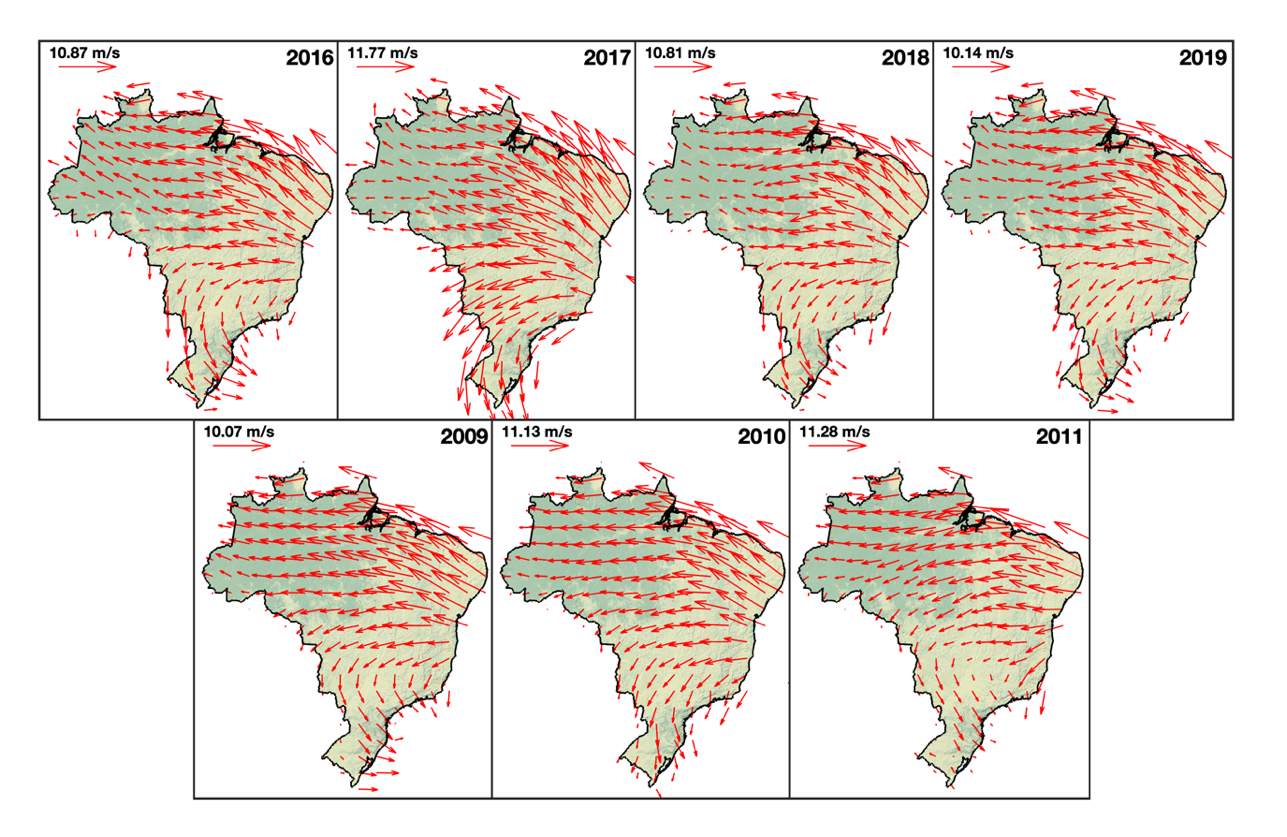
**Figure S1**. July wind fields in emission years of 2016-2019 and sensitivity years of 2009-2011 averaged over 1290 m from surface, max wind speed for scale is given in top left.


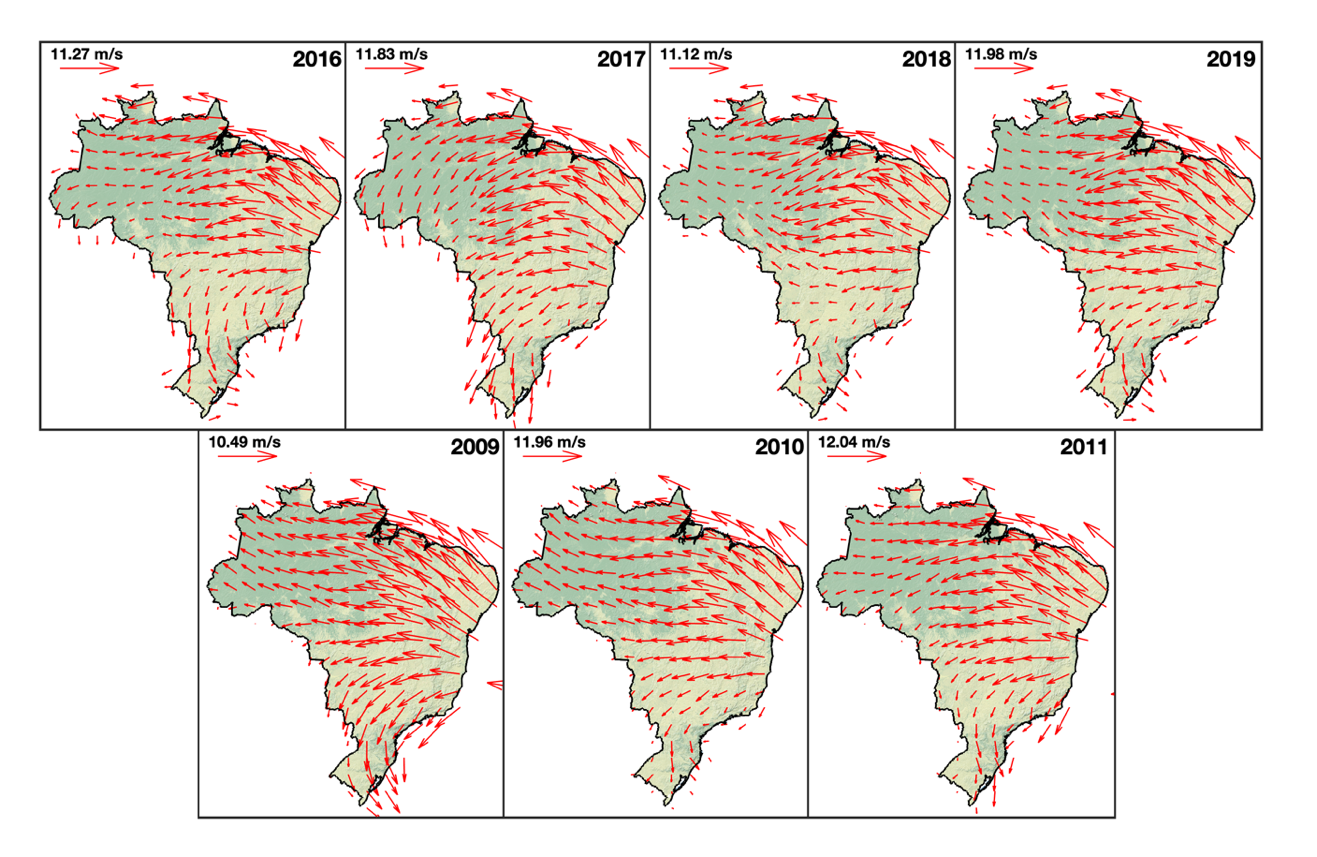
**Figure S2.** August wind fields in emission years of 2016-2019 and sensitivity years of 2009-2011 averaged over 1290 m from surface, max wind speed for scale is given in top left.


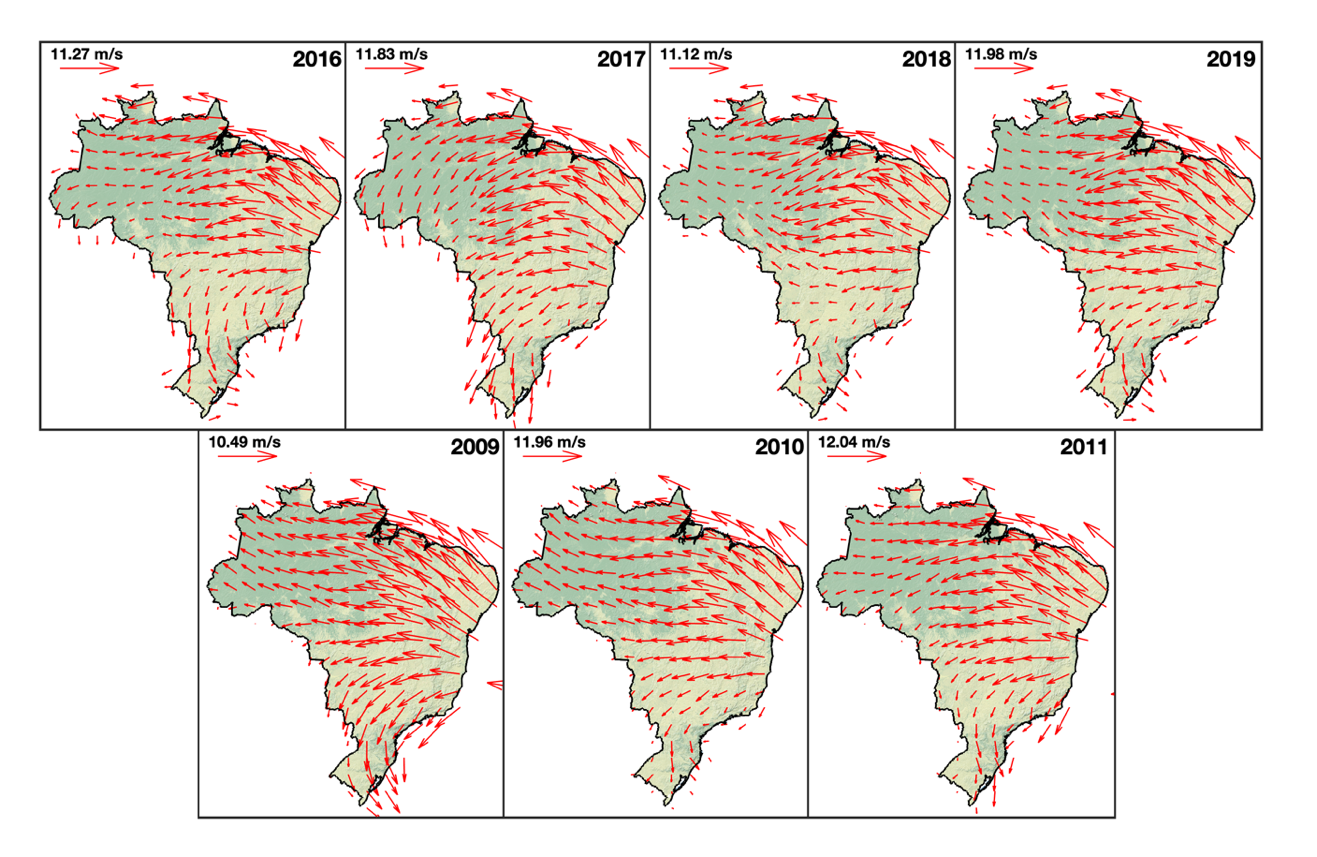
Figure S3. September wind fields in emission years of 2016-2019 and sensitivity years of 2009-2011 averaged over 1290 m from surface, max wind speed for scale is given in top left.

| **YEAR** | **FINN** | | **QFED** | |
| --- | --- | --- | --- | --- |
|  | **BC** | **OC** | **BC** | **OC** |
| **2016** | 52.1% | 52.5% | 56.4% | 54.9% |
| **2017** | 60.8% | 61.0% | 61.2% | 59.5% |
| **2018** | 58.7% | 59.1% | 56.8% | 55.6% |
| **2019** | 61.8% | 62.0% | 62.8% | 59.3% |
| **4YAV** | 58.3% | 58.7% | 59.3% | 57.3% |

Table S1. Percentage of total annual emissions occurring in the three month study period (July-September) for all years and across both species and emission inventories.

| **LAND TYPE** | **EMISSIONS (Gg)** | | | | | **PREMATURE DEATHS** | | | | |
| --- | --- | --- | --- | --- | --- | --- | --- | --- | --- | --- |
|  | 2016 | 2017 | 2018 | 2019 | MEAN | 2016 | 2017 | 2018 | 2019 | MEAN |
| **Evergreen Broadleaf Forest** | 556.1 | 754.3 | 489.6 | 1048.1 | 712.0 | 477.4 | 580.9 | 368.3 | 659.9 | 521.6 |
| **Deciduous Needle Leaf Forest** | 113.5 | 151.8 | 91.3 | 195.1 | 137.9 | 213.2 | 238.3 | 139.1 | 226.8 | 204.4 |
| **Deciduous Broadleaf Forest** | 74.1 | 101.5 | 57.1 | 126.6 | 89.8 | 168.0 | 191.0 | 105.5 | 175.2 | 159.9 |
| **Mixed Forest** | 63.2 | 82.8 | 47.1 | 103.4 | 74.1 | 156.2 | 174.8 | 97.4 | 165.0 | 148.3 |
| **Closed Shrubland** | 59.7 | 81.4 | 44.2 | 98.6 | 71.0 | 155.7 | 178.8 | 97.6 | 172.4 | 151.1 |
| **Open Shrubland** | 60.1 | 85.5 | 42.9 | 100.4 | 72.2 | 178.0 | 202.1 | 105.4 | 191.3 | 169.2 |
| **Woody Savannas** | 87.9 | 129.7 | 60.7 | 156.3 | 108.6 | 254.1 | 298.6 | 149.8 | 308.3 | 252.7 |
| **Savannas** | 381.8 | 537.7 | 253.6 | 512.0 | 421.3 | 1272.3 | 1512.8 | 762.1 | 1504.5 | 1262.9 |
| **Grassland** | 87.4 | 108.5 | 50.0 | 120.9 | 91.7 | 566.6 | 657.6 | 335.0 | 596.8 | 539.0 |
| **Permanent Wetlands** | 33.3 | 41.7 | 21.8 | 46.9 | 36.0 | 331.5 | 395.5 | 213.1 | 319.1 | 314.8 |
| **Croplands** | 23.1 | 29.8 | 15.7 | 28.6 | 24.3 | 324.4 | 396.6 | 213.6 | 294.2 | 307.2 |
| **Urban** | 10.5 | 13.9 | 7.4 | 12.3 | 11.0 | 192.2 | 246.0 | 136.4 | 173.0 | 186.9 |
| **Other** | 13.1 | 12.9 | 9.7 | 12.5 | 10.9 | 245.0 | 199.9 | 133.4 | 179.1 | 189.4 |

Table S2. Emissions and premature death contributions by MODIS land type.
